# A practical guide for selecting continuous monitoring wearable devices for community-dwelling adults

**DOI:** 10.1016/j.heliyon.2024.e33488

**Published:** 2024-06-22

**Authors:** Jessica K. Lu, Weilan Wang, Jorming Goh, Andrea B. Maier

**Affiliations:** a Centre for Healthy Longevity, National University Health System, Singapore, Singapore; b Healthy Longevity Translational Research Program, Yong Loo Lin School of Medicine, National University of Singapore, Singapore, Singapore; c Department of Physiology, Yong Loo Lin School of Medicine, National University of Singapore, Singapore, Singapore; d Department of Human Movement Sciences, @AgeAmsterdam, Faculty of Behavioural and Movement Sciences, Vrije Universiteit Amsterdam, Amsterdam Movement Sciences, the Netherlands

**Keywords:** Wearable devices, Digital health, Physiological monitoring, Validation, User-centered design

## Abstract

**Importance:**

The burgeoning landscape of wearable devices warrants a guide for the selection of devices. Existing guidelines and recommendations provide evaluation frameworks with theoretical principles but tend to lack a pragmatic application and systematic approach for device selection. While fitness trackers exemplify the convenience of wearable technologies, their selection for specific health monitoring purposes demands a nuanced understanding of varying functionalities and user compatibilities.

**Objective:**

The objective is to develop and present a practical guide for researchers, healthcare professionals, and device users to systematically select wearable devices for continuous monitoring in community-dwelling adults.

**Methods & results:**

Based on diverse sources, such as the United States Food and Drug Administration (FDA), the Clinical Trials Transformation Initiative (CTTI), the Electronic Patient-Reported Outcome (ePRO) Consortium, and comparative analyses of wearable technology performances from feasibility and usability studies, the guide incorporates five core criteria: continuous monitoring capability, device availability and suitability, technical performance (accuracy and precision), feasibility of use, and cost evaluation. The structured criteria can be applied in device selection as well as device evaluation.

**Conclusions:**

This practical guide provides a step-by-step solution for researchers, healthcare professionals, and device users to choose suitable wearable devices for continuous monitoring. It provides a comprehensive starting point, outlining how to effectively navigate the selection process for wearable devices amidst a plethora of similar options.

## Highlights

• A step-by-step practical guide to select wearable devices for community-dwelling adults.
• It can aid researchers, healthcare professionals, and device users.
• Five criteria: continuous monitoring, availability and suitability, accuracy and precision, feasibility, and cost.
• Guidelines from the United States Food and Drug Administration were incorporated.
• Includes Clinical Trials Transformation Initiative and Electronic Patient-Reported Outcome Consortium recommendations.

## 1 Introduction

Digital health technologies, particularly consumer wearable devices, have emerged as popular tools for monitoring lifestyle habits and health status [1]. These devices, like fitness trackers, offer convenient and cost-effective solutions for remote data collection of mobility and vital signs, which are influenced by daily activities and behaviour [2]. Wearable devices with continuous monitoring technology, such as continuous glucose monitors, are valuable in medical research for consistent data collection outside clinical settings [3]. A variety of measurements are available, such as sleep patterns, physical activity, heart rate, and blood oxygen saturation, that can supplement health assessments conducted during in-clinic visits [4]. The rapid pace of technological advancement produced a plethora of monitoring devices, making selecting a wearable device a daunting task due to the abundance of similar options [5].

The variety of wearable devices has burgeoned in the past decade [5,6], but there is a lack of a practical and user-centric guide for device selection. The choice of wearable device is dependent on the measurement objectives, user population, and available resources. Current frameworks and recommendations, including those from the United States Food and Drug Administration (US FDA) [7], the Clinical Trials Transformation Initiative (CTTI) [8], and the Electronic Patient-Reported Outcome (ePRO) Consortium [9], provide foundational theoretical principles; however, they tend to lack a pragmatic, step-by-step application for researchers, healthcare professionals (HCPs), and device users who have limited knowledge in digital health to select wearable devices for continuous monitoring [10]. Moreover, existing guidelines and evaluation frameworks do not offer a systematic approach to consider verification, validation, feasibility, and protocol design and execution, which are crucial in research and clinical settings or usage in daily life [11].

To address this gap, a practical guide offering a systematic approach with sequential steps and suggested evaluation metrics to selecting wearable devices for community-dwelling adults is developed and presented. It aims to bridge theoretical advice with actionable implementation and aid in navigating device selection.

## 2 Methods

### 2.1 Sources consulted

To construct the selection guide for continuous monitoring wearable devices for use by community-dwelling adults, recommendations from the US FDA [7], the CTTI [8,12], and the ePRO Consortium [9] were consulted. The FDA guidance outlines considerations for digital health technology usage for remote data acquisition in clinical investigations [7]. Finalized in December 2023, their guidance for industry, investigators, and other stakeholders provides an overview of the considerations when using digital health technologies, such as digital health technology selection, regulatory submission, validation, usability, clinical outcome evaluation, statistical analysis, risks, and data management. The CTTI is a group of individuals and organizations with the mission of improving the quality and efficiency of clinical trials. Their recommendations focus on a framework of specifications to consider during digital health technology selection [12] as well as considerations for data capture during clinical trials (e.g., data collection, management, analysis, and interpretation and protocol design and execution) [8]. The ePRO Consortium included scientists and specialists from Critical Path Institute's clinical outcome assessment and their pharmaceutical trial, development, and validation specialists. The recommendations pertained to the selection and evaluation of wearable devices and their measurements for use in regulatory trials [9]. To obtain an understanding of the available evaluation frameworks, the overview by Coravos et al. had been consulted [11]. They identified the source (government, academia, or industry), evaluation type (organizational- or product-level), product audience (pre-market or post-market users), product scope (software or hardware evaluation), and evaluation criteria (verification and validation, data rights and governance, utility and usability, and economic feasibility) of existing frameworks that are used for risk-benefit analyses of connected technologies. They also provided design principles for reference when constructing the current practical guide, such as including objective, observable, and verifiable criteria in an evaluation process that is contextual and multidimensional [11]. While existing frameworks often offer generalized considerations and thresholds, the distinctiveness of this practical guide lies in its provision of a detailed, step-by-step methodology, akin to a recipe. The COnsensus-based Standards for the selection of health Measurement INstruments (COSMIN) Risk of Bias tool was integrated to ensure a procedure for evaluating technical performance and validation was included [13]. To incorporate vital considerations that shape the users' perceptions of device acceptability and usability into the practical guide, comparative analyses of device performance and user experience from feasibility and usability studies [[14], [15], [16], [17], [18], [19]], along with recommendations from the ePRO Consortium, were consulted to identify the parameters for evaluating feasibility of use.

### 2.2 Development of the selection guide

A wearable device is a compact electronic device equipped with sensors that can be worn on the body or integrated into clothing and other body-worn accessories [20]. These devices are able to remotely, non-/minimally-invasively, and longitudinally monitor essential physiological and biochemical measurements [20]. The practical guide comprises five core criteria for selecting wearable devices: continuous monitoring capability, device suitability and availability, technical performance (accuracy and precision), feasibility of use, and cost evaluation. These criteria are sequentially incorporated to evaluate the wearable device for continuous monitoring in community-dwelling adults. At each criterion, the guide gradually helps to define the context for use, which enable a multidimensional evaluation yet systematic selection of a suitable device. Continuous monitoring is important for uninterrupted and consistent data collection of measurements with high variance and to understand an individual's daily activities and behaviour [21]. Ensuring continuous monitoring capability aligns with the goal of using wearable devices to capture health and behaviour longitudinally and passively outside of clinical settings [20]. The device availability and suitability criterion helps to maximize the device's lifetime of usage and considers the logistical aspects of using the device daily [14]. Evaluating this criterion next ensures the relevancy and reliability of the wearable device as well as the suitability for integration into the daily lives of community-dwelling adults [1]. Technical performance (accuracy and precision) facilitates the collection of high-quality data, forming the basis for analytical insights. The reliability and validity of collected data ensures that the outcomes and insights derived are accurate representations of physiology and behaviour [21]. Feasibility of use aims to enhance user experience, promoting high compliance/adherence by ensuring device user-friendliness. This is a critical consideration in the context of device usage by community-dwelling adults, where factors such as user acceptance, device usability, and data relevancy play pivotal roles in the success of monitoring initiatives [22]. This criterion additionally addresses monitoring needs in research and clinical settings, identifying devices that can seamlessly integrate into workflows for researchers and HCPs [3,8,11]. Cost evaluation is crucial for making informed and financially sustainable decisions, ensuring that needs align with financial constraints and economic value can be optimized [11].

In the practical guide, a holistic approach is adopted to evaluate a comprehensive set of criteria, ensuring user-centric design for researchers and HCPs during the device selection process. By prioritizing the feasibility of use as a core criterion, researchers and HCPs can choose wearable devices with the device end-user in mind, additionally ensuring that user needs are understood and incorporated into the device selection process.

## 3 Results

**Fig. 1:**
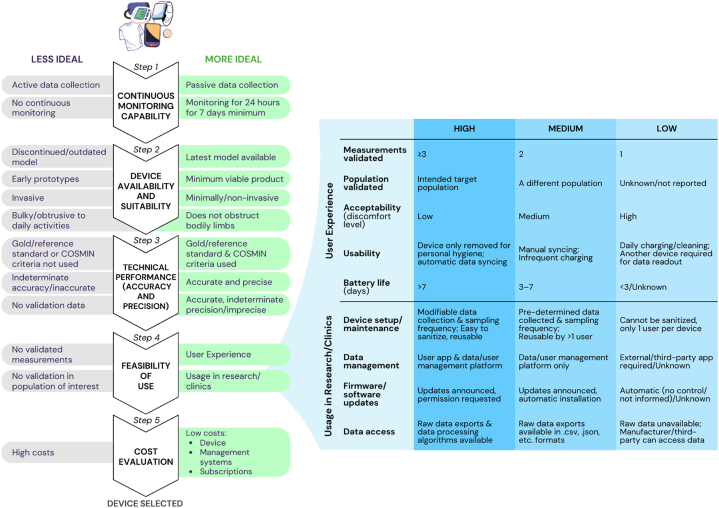
**Guide to select continuous monitoring wearable devices for community-dwelling adults.** Wearable devices are evaluated based on five core criteria: Continuous monitoring capability, Device availability and suitability, Technical performance (accuracy and precision), Feasibility of use, and Cost evaluation. Devices are less ideal or more ideal for further consideration based on the characteristics listed in the grey and green rectangles, respectively. Feasibility is evaluated based on five parameters for User Experience and four parameters for Usage in Research/Clinics. High feasibility indicates what the ideal scenario looks like, medium feasibility indicates that it is less ideal and that some compromise may be required, and low feasibility indicates a scenario that would not be ideal. COSMIN, COnsensus-based Standards for the selection of health Measurement Instruments. (For interpretation of the references to colour in this figure legend, the reader is referred to the Web version of this article.)

The wearable device selection guide (Fig. 1) facilitates the selection and evaluation of continuous monitoring wearable devices for community-dwelling adults. It can be used for selection from a list of devices or for evaluation of one specific device. Detailed definitions and descriptions for how to evaluate each criterion are in Table 1.

**Table 1:** Five core evaluation criteria to select continuous monitoring wearable devices for community-dwelling adults.

Criteria	Definitions and descriptions
1) Continuous monitoring capability	•Passive measurement and data collection for 24 hours for seven days minimum to capture sufficient data of daily activities and behaviour [14], while device can be removed for cleaning or charging
2) Device availability and suitability	•Availability: latest device models commercially available (i.e., not discontinued or outdated) from the original manufacturer; devices in their various stages of development can be considered depending on user and study requirements (e.g., minimum viable products) •Suitability: devices are non-invasive, not bulky, and do not interfere with activities of daily living [17] o Minimally invasive devices (e.g., continuous glucose monitors) can be considered if accepted by user or study requirements
3) Technical performance (accuracy and precision)	•Validation data can be obtained from peer-reviewed articles and specifications published by the device manufacturer •Validation studies should use gold/reference standards and COSMIN criteria to validate technical performance •Accuracy (measurement error) o COSMIN validation measures: standard error of measurement (SEm), smallest detectable change (SDC), limits of agreement (LoA), or coefficient of variation (CV) for continuous scores; % specific agreement (sensitivity (SE)/specificity (SP)/positive predictive value (PPV)/negative predictive value (NPV)) for dichotomous/nominal/ordinal scores o Sufficient accuracy: [SDC or LoA or 95% confidence interval of CV (i.e., CV$\times \sqrt{2\times 1.96\;}$)] < Minimal Important Change (MIC) [13], % specific agreement > 80% [13], or determined from conclusions in validation studies • Precision (reliability) o COSMIN validation measures: intraclass correlation coefficient (ICC), Lin’s concordance correlation coefficient (CCC), Pearson’s correlation coefficient (r), or Spearman’s correlation coefficient (ρ) for continuous scores; Cohen’s (weighted) kappa (κ) for dichotomous/nominal/ordinal scores o Sufficient precision: ICC or (weighted) kappa ≥ 0.70 [13] • International guidelines (e.g., AAMI/ESH/ISO or BHS for blood pressure) other than COSMIN criteria should be used if available
4) Feasibility of use	• User experience: for user to wear device continuously and consistently [15] o Measurements *validated*: increased convenience if one device collects more validated measurements o Population *validated*: ideally validated in an intended or similar population of interest o Acceptability: ideally lower discomfort for user [14]; may include considerations such as form factor [11, 26], material quality [11, 22], thermal management [11], etc. o *Usability*: ease of use of device based on interaction level required between user and device (e.g., maintenance cleaning of device, device removal only for personal hygiene, automatic/manual syncing/uploading data, charging of device, and whether another device is required for data readout) [15, 25] o *Battery life* (after one charge): ideally greater than seven days [14] for infrequent charging to reduce user burden [19]; charging a device daily is least ideal [14] • Usage in research and/or clinical practice: important for protocol design, execution of operations, data analysis, and interpretation o *Device setup/maintenance*: easy device user account creation, modifiable data collection and sampling frequency, easy to sanitize/water-resistant, and reusable by more than one user; may consider device/operating system compatibilities for user or study integrations with other devices and systems [23] o *Data management*: user-interactive app and data/user management platform for researcher/HCP provided by device manufacturer or an external/third-party app/software required; may include considerations for technical support from manufacturer (e.g., troubleshooting, online/offline data transfer, data transmission latency via Bluetooth/Wi-Fi/cable, etc.) and data security and governance (e.g., end-user license agreements and terms of service agreements) [11, 7] o *Firmware/software updates*: updates to firmware and/or software that change raw data collection/processing can be controlled by researcher/HCP [16] o *Data access*: data and processing algorithms are accessible to/shared with researcher/HCP; security/privacy controls managed by researcher/HCP; raw data are available and can be exported in various formats (e.g., .csv, .json, etc.) [23]
5) Cost evaluation	• Lower cost is ideal for devices, related management systems, usage subscriptions, and/or software/platform license fees to researcher/HCP or device user for data storage and analysis

Abbreviations: AAMI/ESH/ISO, United States Association for the Advancement of Medical Instrumentation/European Society of Hypertension/International Organization for Standardization; BHS, British Hypertension Society; COSMIN, COnsensus-based Standards for the selection of health Measurement Instruments; HCP, healthcare professional.

### 3.1 Criterion 1: continuous monitoring capability

Adapted from FDA guidance [7], this criterion selects devices capable of continuous monitoring. If devices do not facilitate passive measurement (i.e., without user interaction) and data collection over 24 hours a day for seven days minimum, they are less ideal as devices without continuous monitoring are not considered in this guide.

### 3.2 Criterion 2: device availability and suitability

The evaluation of the availability and suitability criterion is derived from recommendations from the ePRO Consortium [9]. The availability of a device (discontinuation of or obsolete (i.e., outdated) models and stage in the device development cycle) and its suitability (such as invasiveness, bulkiness, and interference with activities of daily living [17]) will affect the logistics and operations of using the device for researchers, HCPs, and device users.

### 3.3 Criterion 3: technical performance (accuracy and precision)

The third criterion evaluates devices for the accuracy (measurement error) and precision (reliability) of measurements of interest. Examples of measurements include cardiometabolic, neurological, activity and sleep, and psychological biomarkers [25]. This criterion is based on guidance from the FDA [7], recommendations from the CTTI [12], and the verification, analytical validation, and clinical validation (V3) framework [16]. This criterion utilizes the COSMIN Risk of Bias tool for the definitions of sufficient accuracy and precision [13]. International guidelines (e.g., US Association for the Advancement of Medical Instrumentation/European Society of Hypertension/International Organization for Standardization (AAMI/ESH/ISO) or British Hypertension Society guidelines for blood pressure measuring devices) should be used if available. Using validation data from peer-reviewed articles and usage and specifications information from the manufacturer are recommended during device evaluation [11]. Devices can be considered if they are accurate and precise or accurate with indeterminate precision/imprecise. Devices are not considered if the gold/reference standard and COSMIN criteria were not used, or the measurement of interest was not validated.

### 3.4 Criterion 4: feasibility of use

Combining insights from usability studies [14,15,17,18] with the CTTI recommendations [12], this criterion ensures the device selected would be user-centric and user-friendly based on five parameters (measurements validated, population validated, acceptability, usability, and battery life) and addresses workflow needs in research and clinical settings based on four parameters (device setup/maintenance, data management, firmware/software updates, and data access) (Fig. 1). Each feasibility parameter is categorised into high, medium, or low feasibility for usage of the device to provide a relative scale for comparison.

### 3.5 Criterion 5: cost evaluation

Built upon recommendations from the CTTI and Coravos et al., this criterion considers economic aspects for making informed decisions in device selection [11,12]. Possible costs include the costs for the device, for related management systems, for usage subscriptions, and/or for software platform licenses.

**Fig. 2:**
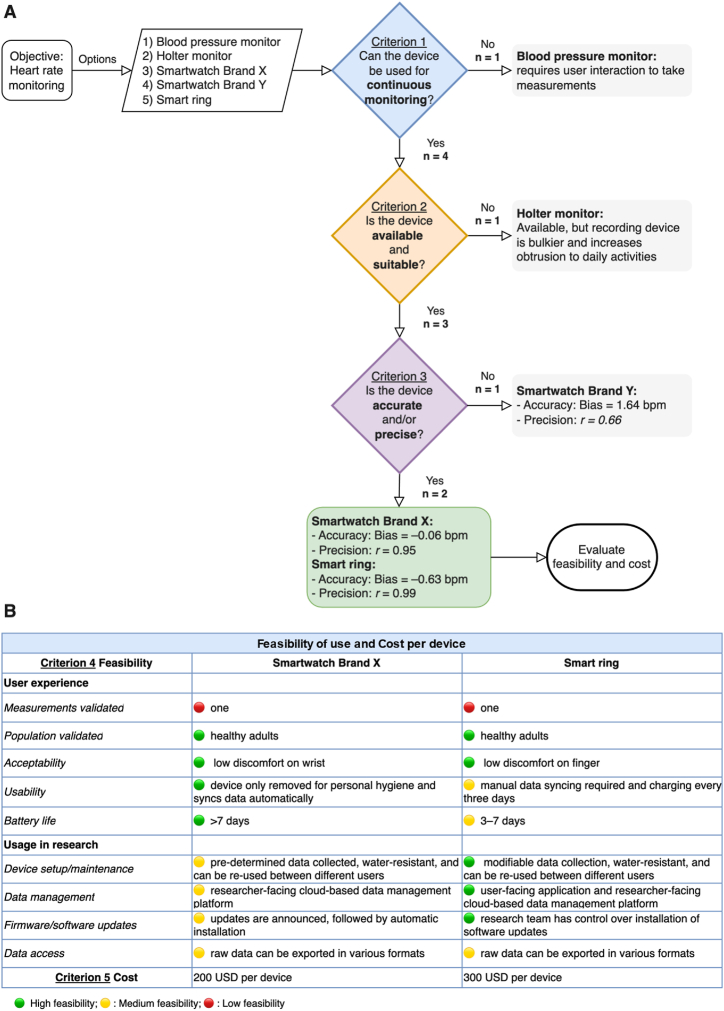
**Example of the wearable device selection process for heart rate monitoring. (A)** The objective is to monitor heart rate, with five device options: an oscillometric automatic blood pressure monitor, a Holter monitor, two smartwatches with optical heart rate sensors, one from Brand X and one from Brand Y, and a smart ring with an optical heart rate sensor. The options are assumed to be representative of the types of devices available on the market for illustrative purposes. The blood pressure monitor is excluded as it cannot provide continuous monitoring. The Holter monitor is excluded due to its bulkier recording device, making it unsuitable for daily use. Based on validation studies in peer-reviewed articles, if the device heart rate measurement is inaccurate, then it would be excluded (e.g., smartwatch Brand Y). Smartwatch Brand X and the smart ring are included in the next evaluation phase as their heart rate measurements are accurate. **(B)** Each feasibility parameter is evaluated and rated as high, medium, or low feasibility, with explanations provided. The cost per device is included for a comprehensive assessment. Although the parameters are not linearly additive, summing the number of high feasibility ratings (green circles) can serve as a proxy for identifying a suitable continuous monitoring device. In this example, despite the higher cost, the smart ring demonstrates the highest feasibility for continuous heart rate monitoring in a healthy adult population. For clarity and brevity, device details and the selection process have been simplified. (For interpretation of the references to colour in this figure legend, the reader is referred to the Web version of this article.)

Fig. 2 presents a step-by-step example of the wearable device selection process using hypothetical devices for heart rate monitoring. The flowchart illustrates decision-making at each criterion, starting with the exclusion of unsuitable devices (Fig. 2A) and culminating in the evaluation of feasibility parameters and cost (Fig. 2B). This example simplifies the details and characteristics of the devices and steps for clarity, providing a clear and practical application of the guide.

## 4 Discussion

A practical guide for selecting wearable devices for continuous monitoring in community-dwelling adults was developed, comprising five core criteria: continuous monitoring capability, device availability and suitability, technical performance (accuracy and precision), feasibility, and cost evaluation. The guide can be applied both to select a device from a list of potential devices and to evaluate a single device.

Existing guidelines, including the FDA draft guidance [7], the CTTI framework [12], and recommendations from the ePRO Consortium [9], served as foundational sources to establish this guide. Existing guidelines focused on usage of digital health technologies in clinical investigations [7] and clinical trials [8]. Hence, they lack practical application and selection considerations for use in community-dwelling adults. Recommendations from the ePRO Consortium provided a sequential list of factors influencing device suitability [9] in regulatory clinical trials and may, therefore, be inadequate to use for other indications or selecting a device for continuous monitoring in daily life. Additionally, these guidelines all have different definitions of digital health technologies, some including digital applications, wearable devices, and other portable technologies containing sensors for remote data acquisition [8], all of which may have different selection criteria. Meanwhile, this practical guide recommends a systematic yet adaptable approach that caters specifically to the selection of wearable devices for continuous monitoring in community-dwelling adults.

This practical guide is designed to enable researchers, HCPs, and device users to identify what may be suitable for continuous and longitudinal monitoring using a systematic process. However, this is a suggested systematic process because depending on the monitoring objectives, user population, and available resources, users of the guide can put weighted emphasis or importance on different criteria. For example, the requirement for some core criteria could be valuing and evaluating feasibility before technical performance (e.g., if the user population entails higher acceptability and usability of the device with a longer battery life) or prioritize costs as the paramount deciding factor (e.g., if financial resources are limited). Data management and device setup/maintenance are essential parameters for researchers and HCPs to evaluate and plan for, but it may not be crucial for device users to consider if they do not need to routinely export and clear existing data to set up devices for new users [18]. Nonetheless, using a practical guide can foster clarity and transparency in decision-making, facilitating communication among researchers, HCPs, and device users to identify the best practices for selecting and implementing wearable devices. While the applicability of the practical guide is currently theoretical, empirical validation in the future can verify the effectiveness of the practical guide in real-world settings.

### 4.1 Strengths and limitations

The technological life cycle of digital devices entails rapid obsolescence of hardware and software [26], which is constantly upgraded and redesigned to newer models. One strength of this guide lies in its adaptability to accommodate the evolving landscape of wearable technologies. As new devices and technologies emerge, the guide's criteria can be adapted to accommodate novel innovations and considerations. Additionally, the systematic approach streamlines the device selection process and minimizes the risk of selecting inappropriate devices, thereby optimizing resource allocation. Furthermore, the guide's considerations of both technical performance and user experience promotes the selection of devices that are not only accurate but also well-received by users, which facilitates high compliance/adherence. From a technological innovation perspective, the practical guide can also serve as a reference point or benchmark for device designers and manufacturers/engineers, providing suggestions for evaluation components of user-friendly devices and improvements to hardware and software to enhance data quality.

It is noted that device selection considerations out of scope are social/psychological factors that could translate to cultural preferences that may affect device acceptability and usability [14]. Social/psychological factors should be incorporated into the device selection process according to specific monitoring needs and circumstances of the intended target population. Additionally, the guide relies on the availability of validation data from peer-reviewed articles as well as usage and specifications information from the manufacturer. These validation data ensure a fair comparison across devices; however, they may not always be readily accessible. While the guide's criteria are modular and can be adapted to address novel innovations and considerations, there remains the challenge of who will update the guide and how regularly it will be updated in response to the rapid technological advancements in wearable devices.

Future directions include validating this practical guide by disseminating the guide to users and obtaining feedback on the usability of the guide. Various research teams, healthcare providers, and community-dwelling device users could be invited to form focus groups or a consortium to identify areas of the practical guide for revision and improvement. The feedback and testing would help improve the guide's user-friendliness and applicability in different contexts, increasing the user-centricity of the guide. Moreover, the entire selection process could be developed into a web or mobile application where users can input their measurement objectives and user needs to obtain recommendations of suitable devices. This endeavour may need to be developed and maintained by a non-profit organization with no conflicts of interest or biases towards recommending certain devices.

## 5 Conclusions

Overall, there is an abundance of digital health technologies, and the variety is likely to increase further with the rapid advancement of technologies. A practical guide on the selection of suitable wearable devices for continuous monitoring in community-dwelling adults includes evaluating continuous monitoring, device availability and suitability, technical performance, feasibility, and costs. This guide, while providing a structured approach for device selection, also opens avenues for exploring personalized health monitoring strategies in diverse community settings. The proposed selection process provides a systematic approach that researchers, healthcare professionals, and device users can apply and implement.

## Funding

None.

## Data availability statement

No original data was used for the research described in the article.

## CRediT authorship contribution statement

**Jessica K. Lu:** Writing – review & editing, Writing – original draft, Visualization, Methodology, Formal analysis, Conceptualization. **Weilan Wang:** Writing – review & editing, Methodology, Conceptualization. **Jorming Goh:** Writing – review & editing, Supervision, Conceptualization. **Andrea B. Maier:** Writing – review & editing, Supervision, Conceptualization.

## Declaration of competing interest

The authors declare that they have no known competing financial interests or personal relationships that could have appeared to influence the work reported in this paper.
